# Supplementary material for: Are carbon emissions trading and green financial instruments synergistic? -Comprehensive quantitative research based on content analysis
Source: PLoS One. 2024 Mar 7;19(3):e0298601. doi: 10.1371/journal.pone.0298601 (PMC10919601; doi:10.1371/journal.pone.0298601)
Supplement: S1 Data — (ZIP) [file pone.0298601.s001.zip › Data/地级市Z_F工作报告.zip!/╣π╬≈/┴°╓▌╩╨/└┤╫╘╣1⁄2╓┌║┼í╛┬φ┐╦╩2╛▌═°í┐_2011.docx]

各位代表：

　　现在，我代表市人民政府向大会报告工作，请予审议，并请各位政协委员和列席人员提出意见。

2010年工作回顾

　　过去一年，在自治区党委、政府和市委的正确领导下，全市上下深入贯彻落实科学发展观，按照市委提出的“三十字方针”，扎实推进“三个同步”，全面实施“二次创业”，大力开展“七个年”活动，努力克服百年一遇的特大干旱和严重洪涝灾害等困难，加快“经济升级，城市转型”步伐，全面超额完成了市十二届人大八次会议确定的目标任务。

　　初步统计，全市实现地区生产总值1260.3亿元，增长16.0%；工业总产值2652.6亿元，增长31.5%；全社会固定资产投资1005.1亿元，增长47.4%；财政收入201.2亿元，增长27.6%。城镇居民人均可支配收入17715元，增长10.6%；农民人均纯收入4945元，增长14.2%。城镇登记失业率控制在4.5%以内。人口自然增长率为6.96‰。为民办20件实事基本完成。我市成为全国唯一独揽“2010中国最具创新力城市”、“2010中国经济转型示范城市”和“中国最具竞争力特色经济市”三项殊荣的城市。

　　“十一五”规划目标全面实现。过去的五年，是我市历史上发展最好、最快的时期之一。与2005年比，全市地区生产总值增长了1.1倍，年均增长15.4%；工业总产值增长了2.1倍，年均增长25.7%；全社会固定资产投资增长了4.9倍，年均增长42.4%；财政收入增长了1.51倍，年均增长20.2%。城镇居民人均可支配收入年均增长13.1%，农民人均纯收入年均增长14.3%。三次产业比重由2005年的11.5:52.0:36.5发展为8.3:63.0:28.7。市区面积由原658平方公里扩大为1016.75平方公里。在全区率先实现工业化示范城市的奋斗目标基本完成。

　　一、“三年四千亿，工业再翻番”迈出坚实一步，工业经济规模、质量和效益显著提升

　　工业发展实现五大突破。一是规模以上工业总产值突破2000亿大关，达2387.7亿元，增长33.3%。二是工业利、税双双突破百亿大关，利税、利润总额分别达216.5亿元和108.1亿元?，增长35.0%和54.9%，创下历史最好水平。三是汽车工业产值突破千亿大关，达到1027亿元，成为我市首个、全区第二个千亿元产业。四是企业发展取得新突破，柳钢主营业务收入超过450亿元，上汽通用五菱突破400亿元?，东风柳汽突破100亿元，全市百亿元企业增至5家；产值过亿元企业由200家增至260家；新增规模以上工业企业125家，总数达984家。五是县区工业总产值突破1000亿元，柳南区、柳北区工业产值分别突破200亿元?。

　　产业结构优化升级加快。工业更新改造投资287.5亿元，增长37.9%。支柱产业不断壮大，全市各类汽车产量143.7万辆，增长15.5%；钢和钢材产量双双突破1000万吨，柳钢跻身中国千万吨钢企行列；工程机械产量突破5万台，柳工轮式装载机销量保持国际第一。培育新支柱产业初见成效，化工产业产值突破100亿元，柳化完成六大生产基地布局，成为亚洲最大的保险粉和国内最大的双氧水生产企业。传统优势产业改造升级加快。新兴产业快速发展，新能源汽车、热电联产等项目加快推进。

　　自主创新能力不断增强。第四轮创新计划全面完成。柳州高新区成为国家级高新区。柳工成功获得广西首个国家级企业工程技术研究中心。新增国家级高新技术企业19家，自治区千亿元产业重要研发中心6个，自治区级创新型试点企业10家、工程技术研究中心3个、企业技术中心6个。自主品牌打造取得重大进展。上汽通用五菱“五菱之光”荣获广西科技进步特别贡献奖，企业首个自主品牌轿车“宝骏”成功下线，填补了广西生产中级轿车的历史空白。东风柳汽B12轿车研发取得关键性突破，风行景逸引发两厢车市“鲶鱼效应”。产学研合作不断加强，广西首家“院士籍”企业和首家科技风险投资企业落户柳州。我市率先在全区构建起了区、市、县三级人才小高地体系。

　　园区经济强劲增长。重点监测的八个工业园区规模以上工业产值突破800亿元，达827.7亿元，增长46.5%。园区集聚效应日渐显现，阳和工业新区工业产值突破200亿元，全市产值过百亿工业园区增至5个，自治区级A类以上产业园区由7个增至8个。阳和成为国家新型工业化汽车产业示范基地主导园区。广西柳州汽车城项目正式落户柳东新区。

　　节能减排成果显著。“十一五”节能减排目标任务全面完成。初步核算，单位生产总值能耗比2005年下降22%，二氧化硫、化学需氧量排放分别下降34.5%和13.1%。“十一五”期重点能耗企业总节能量超过300万吨标准煤。

　　二、城乡统筹发展新格局战略扎实推进，县域经济持续快速发展

　　统筹城乡基础设施建设力度加大。建成农村公路965.2公里，修建村屯道路249条660公里，全市乡镇公路通畅率和行政村公路通达率均达100％。农田水利基本建设投资9.7亿元，创历史新高，完成病险水库除险加固53座，新增恢复改善农田灌溉面积10万亩；建成农村饮水工程366处，解决了17.3万农村人口的饮水安全问题。社会主义新农村建设扎实推进。第三批整村推进扶贫开发工作圆满完成。生态家园建设有效开展，农村卫生厕所普及率达52%。农村危房改造、城乡风貌改造年度任务全部完成。农村土地综合整治以及城乡建设用地增减挂钩工作顺利推进。

　　“十大农业工程”顺利推进。工程累计完成投资7.9亿元。良种扩繁工程建成糖蔗良种基地456亩、油茶良种育苗基地640亩、柑橘无病毒苗木繁育基地120亩，引进原种猪1280头；产业基地工程建成优质油茶基地23.1万亩、超级稻基地38万亩、双季葡萄基地3.5万亩、茶叶标准化生产基地5510亩、千亩特色水产养殖基地640亩；现代农业科技示范园、农业服务体系完善等工程加快推进，有力带动了农业发展和农民增收。

　　现代农业稳步发展。全市财政农业投入16.8亿元，增长17.3%。市财政对农业生产性投入在2009年基础上翻了一番。全市农林牧渔业总产值165.2亿元，增长5.3%。面对百年一遇的特大干旱，市政府承诺对稻种损失予以全额补偿鼓励农民按农时进行春播，确保了全年播种面积和粮食产量的稳定。糖料蔗、桑蚕、蔬菜、水果、茶叶等优势产业实现量效并增。农业产业化进程加快，新增市级重点龙头企业12家，总数增至64家；新增农民专业合作社217家，总数达627家；新建标准化规模养殖场163家，规模养殖户达3041户，柳州宏华牧业公司成为全国标准化规模养殖示范场。冬季葡萄面积不断扩大，我市成为全国最大的冬季葡萄生产基地。柳城成为广西第一个出口水果质量安全示范区。农机创新示范工程带动明显，我市水稻耕种收综合机械化水平提升到51%，进入全区先进行列。

　　县域经济快速发展。六县实现地区生产总值394.0亿元，增长15.2%；规模以上工业总产值352.1亿元，增长35.6%；全社会固定资产投资445.9亿元，增长52.2%；财政收入26.3亿元，增长32.0%。鹿寨县地区生产总值突破100亿元，成为继柳江后我市第二个生产总值百亿县。

　 三、服务业发展加快，城市综合服务功能持续增强

　　“十大商贸工程”进展顺利。千亩农贸物流园、千亩钢铁物流园、千亩化工物流园开工建设。新风时代购物广场、桂中海迅柳北物流基地建设顺利推进。成功引进台湾大润发大型超市。建成预备四星级酒店一家，按五星级标准建设酒店一家。商业布局不断完善，时尚休闲、精品购物特色商业街及柳北文化商业广场建成开业；3个商业网点示范社区顺利建成，50个微型超市进入社区。柳侯、桂中等7个集贸市场完成改造。

　　城乡居民消费不断扩大。家电、汽车摩托车下乡及以旧换新政策全面落实，有力拉动了农村消费。“万村千乡市场工程”、“新农村现代物流工程”累计建成农家店1289个，配送中心7个，农村流通网络已覆盖所有乡镇及87%的行政村。升级改造乡镇屠宰场52个，建成放心肉连锁店20家。价格调控监管得到加强，果断依法查处年初部分米粉生产厂家集体涨价事件，得到了市民广泛支持。我市市场供应和物价总水平保持稳定。全市社会消费品零售总额480.0亿元，增长20.0%。

　　“旅游名城”建设加快推进。“十大旅游工程”年度完成投资10.9亿元。马鹿山奇石休闲公园主展馆、“东堤春晓”特色街建设基本完工，市区沿江景观改造、凤凰河休闲度假区等项目顺利推进。龙潭公园成为继柳侯公园之后广西第二个国家重点公园。三江丹洲古城、融水贝江景区成为国家4A级景区，鹿寨月岛湖度假区、君武森林公园等四个景区成为国家3A级景区。成功举办第六届柳州国际奇石节。全年接待过夜入境旅游者8.1万人次，增长19%。

　　房地产业稳定发展。房地产价格监测、市场调控和土地供应批后监管卓有成效，房地产投资与消费保持稳定。全年房地产开发投资165.4亿元，增长37.7%；房屋施工面积1443.1万平方米，增长16.5%；销售面积291.9万平方米，增长9.0%；市区商品房平均价格为4771元/方米，增长11.2%。

　　四、宜居城市建设力度加大，城市承载能力不断提升

　　提升交通枢纽地位工作有效开展。湘桂铁路扩能、柳南城际铁路等项目加快建设。柳肇铁路已向国家发改委申请立项。柳韶铁路规划通过专家组预审。三江至柳州、阳朔至鹿寨、柳州至武宣等高速公路项目顺利推进，柳州至梧州、柳州至南宁第二高速公路列入广西高速公路网规划近期重点建设项目。柳州港总体规划获自治区批准。鹧鸪江码头、阳和码头和旅游客运码头开工建设。落久水利枢纽项目建议书通过水利部水规总院审查，洋溪水利枢纽列入柳江综合规划修编。白莲机场扩建正式启动。

　　城市建设力度进一步加大。市政重点工程完成投资143.3亿元，增长42.3%；其中“十大建设工程”完成投资80.0亿元。城市路网建设加快推进，四座跨江大桥和外环路建设以及三个交叉路口的立交改造进展顺利，一批城市道路建设完工，15条小街小巷完成改造。静兰片区改造扎实推进。城市东扩攻坚战全面打响。“柳东新区十大项目”相继启动，市区至新区快速公交系统（BRT）、柳东三甲医院、实验中学、小学等项目加快推进。新区基础设施建设全面提速，市委党校顺利搬迁，柳东大厦主体竣工，新区路网工程和各安置小区加快建设。广西柳州汽车城建设规划、新区城市规划编制初步完成，各项工作紧密推进。

　　生态建设再结硕果。我市荣获“中国人居环境范例奖”。国家森林城市、国家环境保护模范城市创建工作积极开展。龙潭、雀山等公园一期改造基本完工，一批街头小游园建成使用。“百万人种百万棵树”超额完成，全市绿化造林31.5万亩，我市森林覆盖率达62.6%。城市绿地景观提升、石山河岸绿化、夜景光亮等工程扎实推进，一批道路完成绿化景观改造。六县县城污水及生活垃圾处理设施建成使用，市区污水集中处理率、生活垃圾无害化处理率分别达91%和100%，六县县城分别达68.9%和60%以上。柳江河饮用水保护河段保持国家地表水Ⅲ类水质标准，市区空气质量优良天数354天，优良率为97.0%。

　　城市规划和管理水平进一步提升。《柳州市城市总体规划（2010-2020）》和《柳州市土地利用总体规划（2006-2020）》获得国务院批准。市区控制性详规覆盖率达100%。六县乡镇和村屯规划覆盖率分别达100%和20%。土地宏观调控和矿产资源保护卓有成效。全市新增建设用地指标2334公顷，增长30.9%；盘活存量土地1105公顷，增长64%；“两违”治理拆除违法建筑50.1万平方米。城乡清洁工程和治脏、治乱、治水等专项整治行动深入开展，桂柳路大型垃圾转运站等环卫设施加快建设，市容环境卫生管理水平不断提升。数字城管一期工程建成并实现与“天网工程”同台共享。

　 五、民生持续改善，社会保障水平不断提高

　　创业、就业体系不断完善。创建“国家级创业型城市”扎实推进。“全民创业提升年”活动有效开展，涌现了一批优秀创业典型和创业孵化基地，全年新增创业人数2.02万人。统筹城乡就业取得新进展，市、县、乡、村四级公共就业服务联网初步形成，乡镇（街道）、社区公共就业服务机构组建率分别达100％和93%以上。农村劳动力培训和转移就业力度持续加大。全市新增城镇就业5.4万人，新增农村劳动力转移就业5.6万人。

　　社会保障能力持续增强。社会保险覆盖面不断扩大。企业退休人员基本养老金继续提高。柳江县新型农村社会养老保险试点正式启动，被征地农民养老保障扩面工作顺利开展。市区农村低保标准由1200元提高到1500元。“五缓四降三补贴两协商”继续执行。集体关闭破产企业和困难企业退休人员纳入城镇职工基本医疗保险保障范围。老工伤人员纳入工伤保险统筹管理。“五险合一”、一票征收在全区率先实行。社会救助救灾工作有效开展，灾害应急救援能力不断增强，1456户水毁民房重建全部完成。水库移民生产生活条件得到改善。残疾人事业健康发展。

　　“十大民生改善工程”深入民心。改制企业职工危旧房集中区改造完成投资18亿元，改造面积110万平方米。保障性安居工程建设经济适用房98万平方米，建成廉租房4960套25.8万平方米，筹集、开工建设公共租赁房1248套，发放廉租住房租赁补贴6228户，最低收入家庭廉租住房保障实现“应保尽保”。贫困地区中小学免费午餐范围扩大到柳城县古砦仫佬族乡，共有10.02万学生受益。少数民族村寨防火改造工程全面完成，967个村屯面貌明显改善，火灾事故、死亡人数分别比工程实施前下降了55%和80%。年内少数民族村寨火灾统保工程共为灾民理赔260万元。“老有所养工程”稳步推进，建立100个居家养老试点任务全面完成；市财政补贴1140万元，将老年人免费乘公交车年龄由70岁放宽至68岁，让老年人提前两年享受到了免费公交服务。

　　社会保持和谐稳定。农村“三大纠纷”调处和信访工作不断加强，领导干部公开大接访活动有效开展，社会矛盾得到妥善化解。创建“平安柳州”活动深入推进，“天网工程”二期顺利建成，市区治安监控点增至1440个，“110”接报警案件持续下降，人民群众安全感进一步增强。和谐社区建设扎实推进，建成社区“一站式”服务平台85个。食品药品安全监管机制不断完善。安全生产状况保持稳定好转。

　　六、各项改革稳步推进，对外开放呈现新气象

　　各项改革有力推进。国有及国有控股企业资产总量突破千亿大关，达1100亿元，比2005年翻了两番。政府融资平台公司债务清理核实工作基本完成。工贸与百货合并重组、柳化增资扩股与战略扩张稳步推进。农业农村改革不断深化，集体林权制度改革已完成林地确权发证1166万亩。医药卫生体制改革稳步推进，公立医院改革试点正式启动，基层医疗卫生机构综合改革、实施国家基本药物制度等指标超过自治区要求。文化体制改革扎实推进，市艺术剧院挂牌成立。金融生态环境不断优化，华夏、招商、中信、深圳农商行等四家金融机构入驻柳州；柳州银行正式更名并在南宁、河池、来宾设立分行；融水村镇银行挂牌营业，我市扩大农村金融改革试点走在全区前列。年末金融机构本外币各项存款余额1485.5亿元，贷款余额突破千亿元，达1057.6亿元，分别比年初增长22.0%和22.1%。

　　对外开放呈现新气象。外贸出口呈现恢复性高速增长，全市进出口总值28.2亿美元，增长67.9%；其中出口6.3亿美元，增长72.4%。我市对东盟、美洲、印度市场出口态势良好。出口产品结构不断优化，机电产品占出口总额的58%。企业“走出去”力度持续加大，柳工加快向海外扩张。国际交流与合作不断扩大，成功举办中越青年大联欢系列活动和第二届中国—东盟女企业家创业论坛，全年共接待境外到访团体102批1619人次；先后组团赴印度、墨西哥、巴西等六个国家参加会展或进行经贸对接，进一步提高了柳州国际形象和影响力。

　　招商引资成绩显著。全年签约项目577个，合作金额677.2亿元；区外境内到位资金418.6亿元，增长35.6%。实际利用外资2.4亿美元，增长21.6%，创历年新高。招大引强取得新突破，引进亿元以上重大项目105个，保利集团、中铁物流等相继入驻柳州。区域合作进一步加强，承接东部产业转移合作项目170个，投资金额达196.6亿元。

　 七、社会事业繁荣发展，精神文明建设取得新成绩

　　教育发展水平不断提升。推动城乡义务教育均衡发展，启动新建和扩建11所中小学校，教育布局结构不断优化。素质教育深入实施，中考改革稳步推进。市铁二中、景行小学和柳南区实验小学被评为全国学校艺术教育工作先进单位。普通高中教学质量稳步提高，高考主要指标继续名列全区前茅。学前教育协调发展，幼儿园办园行为进一步规范。特殊教育稳步发展，市特殊教育学校新校园和鹿寨、融安、融水、三江特殊教育学校启动建设。职业教育快速发展，职教园一期建设基本完成，职业技术学院入选国家示范性职业院校；市属5所中等职校成为国家级重点职校，6所成为自治区示范职校，市一职校以全区排名第一入选国家发展改革示范校。投入专项经费3255万元为校园、幼儿园配备专职安全协管员，确保了校园安全。

　　文化、体育事业繁荣发展。“十大文化建设工程”成果显著，水上大舞台竣工并投入使用。重建文庙落成并成功举办祭孔大典。26集动漫剧《心灵之窗》在中央电视台播出，《生日礼物》动画短片荣获两项大奖。电视连续剧《刘三姐》完成拍摄。工业题材大剧《红瑶梦》完成首演。村村通直播卫星覆盖工程圆满完成，解决了6666个边远山区村屯近20万农户收听收看广播电视问题。刘三姐大舞台“周周演”等群众喜闻乐见的文化活动深入开展，“柳江之夏”荣获全国第十五届“群星奖”。非物质文化遗产保护成绩斐然，传统侗戏列入国家名录，民间舞蹈壮族师公舞列入自治区名录。体育事业蓬勃发展，成功举办市第12届运动会和第2届少数民族传统体育运动会，成功承办2010年世界水上极速运动大赛、斯坦科维奇杯洲际篮球赛等国际顶级赛事。组团参加自治区第12届少数民族传统体育运动会和第7届残疾人运动会获得较好成绩。柳州籍运动员在世界体操锦标赛、亚洲运动会等国际赛事屡创佳绩。城乡体育设施不断完善，李宁体育馆和柳州游泳馆基本完工。

　　卫生、人口与计生事业加快发展。工人医院、柳铁中心医院改扩建进展顺利。基本医疗保障制度不断完善，扩大了新农合补偿范围，新农合全市平均参合率达93.66%。实施社区卫生服务机构标准化建设,社区卫生服务能力不断提高。基本公共卫生服务和基本医疗服务基本覆盖城乡居民。艾滋病、手足口病等传染病防控得到加强。全国卫生城市创建活动稳步推进，全民健康教育与控烟活动深入开展。人口与计生工作全面加强，诚信计生工作扎实推进，我市被确定为全国婚育新风进万家活动示范市和全区首批人口计生综合改革示范市。第六次全国人口普查工作取得阶段性胜利。

　　精神文明建设取得新成果。全国文明城市创建深入推进。“柳州市文明形象大使”评选成功举办，“龙城杰出青年卫士”方华被授予“广西青年五四奖章”称号；三级劳模评选及社会主义核心价值体系宣讲系列活动有序开展。我市荣获“自治区未成年人思想道德建设先进城市”称号。城市公共文明指数跃居全国地级市第35位。民族团结宣传教育和民族团结进步创建活动收到良好成效。国防动员和民兵预备役工作成绩突出，双拥共建扎实推进。我市被评为“十一五”全国人民防空先进城市。

　　八、政府建设不断加强，公共服务水平持续提升

　　政务环境进一步改善。全区深化政务公开推进政务服务经验交流现场会在我市召开，柳州经验获得全区推广。政务服务向基层延伸，乡镇（街道）建立政务服务中心、村（社区）建立便民服务站达10%以上。网上审批、并联审批和重点项目绿色通道高效运转，第七轮行政审批项目清理、规范工作顺利推进。公共服务事项进入政务服务中心，更加方便群众办事。“工作落实年”活动扎实开展，绩效考评工作体系和机制不断健全，绩效考评全面推开。坚持市长“企业接待日”制度，帮助一批企业解决了实际困难。

　　党风廉政建设持续加强。“党建引领提升年”活动深入开展。党风廉政建设责任制全面落实，行政监察和审计监督有效开展。纠风治乱工作有序推进。工程建设等关键领域监控机制不断完善，政府投资管理和经济活动日趋规范，源头治理和反腐败惩防体系建设持续加强。

　　民主法治工作有效开展。市政府自觉接受市人大、市政协的监督，广泛听取社会各界意见，认真办理人大代表议案、建议和政协委员提案，答复率达100%。依法治市扎实推进，“五五”普法圆满完成。行动学习项目全面启动，柳南区行动学习卓有成效并成为全区范例。基层群众自治机制不断健全，政务公开、村务公开、厂务公开工作进一步加强。

　　过去一年，我市人事、编制、外事侨务、口岸、工商、税务、技术监督、统计、国家安全、决策咨询、新闻出版、档案、修志、气象、粮食、供销、宗教、老龄、防汛抗旱、防震减灾、社会科学等各项工作都取得了新的成绩。

　　各位代表，2010年，在困难和挑战面前，我们以优异的成绩为“十一五”划上了圆满的句号。这些成绩的取得，是市委正确领导及市人大、市政协监督支持的结果，是全市人民精诚团结、艰苦奋斗和社会各界鼎力支持、积极奉献的结果。在此，我代表市人民政府，向全市人民，向驻柳人民解放军、武警部队官兵，向各民主党派、工商联、人民团体及海内外所有关心和支持我市发展的各界人士，致以崇高的敬意和衷心的感谢！

　　在总结成绩的同时，我们也清醒地看到了存在的不足和问题，主要是：经济结构性矛盾仍然突出，产业结构不尽合理，服务业发展滞后，经济外向度偏低；统筹城乡发展的办法和手段不多，解决“三农”问题任重道远；就业形势依然严峻，部分群众生活还比较困难等等。对此，我们一定高度重视，正确面对，努力解决。

2011年工作任务

　　今年是“十二五”期开局之年，也是实施“三年四千亿，工业再翻番”战略承上启下的一年。刚刚结束的市委十届十四次全会，对我市“十二五”期规划建设和科学发展做出了重大战略部署，为我们描绘了建设更加美好的柳州的宏伟蓝图。做好2011年工作，对于“十二五”期开好头，起好步，对于实现工业再翻番以及建设更加美好的柳州的战略目标，具有重大的意义。

　　今年政府工作的总体要求是：全面贯彻党的十七大和十七届三中、四中、五中全会精神，以邓小平理论和“三个代表”重要思想为指导，以科学发展、转型发展、和谐发展、全面发展为主题，以转变经济发展方式为主线，扎实推进“三个同步”，深入实施“二次创业”，按照市委提出的“科学发展，升级转型；聚集人气，做大城市；努力学习苏州，全面发展柳州”的“二十八字方针”，强力推进“三年四千亿，工业再翻番”战略，着力加快产业结构优化升级，着力提升自主创新能力，着力推动城乡统筹协调发展，着力推进宜居城市建设，着力深化改革扩大开放，着力保障和改善民生、促进社会和谐稳定，进一步巩固和提升柳州“经济升级，城市转型”成果，建设更加美好的柳州。

　　今年我市经济社会发展的主要预期目标是：地区生产总值增长13%以上；规模以上工业总产值增长20%以上；全社会固定资产投资增长15%以上；财政收入增长14%以上；单位生产总值能耗、主要污染物排放控制在自治区下达指标以内；城镇居民人均可支配收入、农民人均纯收入分别增长10%以上；城镇登记失业率控制在4.5%以内；人口自然增长率控制在自治区下达指标以内。

　　为实现上述预期目标，着重抓好以下八项工作：

　　一、深入实施“三年四千亿，工业再翻番”战略，确保工业总产值迈上3000亿元台阶

　　实施经济质量升级规划，加快转变经济发展方式，调整优化产业结构，进一步提升工业经济规模、质量和效益。

　　举全市之力推进广西柳州汽车城建设。全面启动上汽通用五菱40万辆乘用车基地、东风柳汽10万辆商用车基地和25万辆乘用车基地建设，着力打造具有国际水平的现代汽车城。汽车城建设事关柳州长远发展大局，决定城市未来战略地位，必须全面落实汽车城建设总体规划，千方百计确保项目建设用地供给，确保用电、用水、供热、燃气等基础建设同步跟进，确保汽车研发、汽车测试（检测）、汽车交易市场、汽车展示、汽车商务区等配套项目同步启动。要以汽车城建设为契机，强化国家汽车零部件生产基地和国家汽车及零部件出口基地建设，大力实施汽车零部件产业提升战略，加快上汽通用五菱发动机、柳州玉柴20万台柴油机、玲珑轮胎等项目建设，着力引进国内外知名零部件企业，不断完善乘用车、商用车、发动机三大配套体系，推动零部件与整车同步建设、同步发展。今年，全市各类汽车产量达150万辆以上。

　　着力推进工业升级转型。启动“十大工业结构调整工程”，着重推进98个重大工业项目，年内更新改造投资330亿元以上。实施钢铁产品结构调整工程，着力推进板材产品延伸及深加工，大力发展非钢产业。实施装备制造振兴工程，加快柳工液压元件研发制造基地建设，力争建成工程机械驱动桥研发制造基地；提升预应力机械、数控机床制造技术水平。实施化工产业壮大工程，将煤化工产品向盐化工、纸化工方向拓展，加快烧碱、竹浆纸等项目建设，将化工产业逐步培育成新的支柱产业。今年，力争柳钢主营业务收入突破500亿元，柳工突破300亿元，柳化突破100亿元。大力发展新能源与节能环保、机电一体化及电子信息、汽车电子、生物制药等新兴产业，加快银海铝业35万吨铝深加工、鹿寨热电联产等项目建设，积极推进新能源汽车项目建设，着力打造广西新能源汽车产业基地。

　　加快转变经济发展方式。实施“柳州智造”战略，把经济增长动力转换到科技引领、创新驱动的轨道。启动第五轮创新计划和“十大科技提升工程”，推进“三年四千亿”产业提升科技支撑扶持计划200项。加快汽车零部件、循环经济产业技术创新联盟平台和柳州质量技术检测研究中心建设，推进欧维姆国家预应力锚具工程技术研究中心申报工作，力争国家级高新技术企业突破100家，新增自治区级工程技术研究中心3个、企业技术中心3-5个，自治区级产品质量检测中心2个。积极推进工业化与信息化融合，推动一批“两化融合”试点示范项目建设。以我市列为首批国家商标战略实施示范城市为契机，大力推进质量兴市战略、名牌发展战略与技术标准战略，努力争创中国名牌产品和驰名商标。大力发展循环经济，继续推进柳钢、鱼峰水泥等示范单位项目建设，突出抓好重点行业及重点能耗企业的节能降耗，着力打造生态工业柳州。

　　大力扶持中小企业发展。继续实施中小企业成长计划，通过产业引导、政策配套、资金扶持、强化服务等措施，发展壮大一批中小企业，确保产值过亿元企业达280家。实施金融支持百家中小企业健康成长计划，发挥好产业投资公司等融资平台支持功能，鼓励企业通过发行集合债券等方式缓解资金压力；积极引进、发展风险投资和创业投资公司，引导各类资本投向新兴产业。重点推进50个“中小企业和新兴产业科技扶持计划”项目，努力培育一批拥有自主知识产权和自主品牌的科技型中小企业。继续主动承接东部产业转移，加快劳动密集型和技术密集型产业发展。

　　加快发展园区经济。充分发挥政策优势，积极推动柳州高新区发展提速。配合广西柳州汽车城建设，全面加快阳和工业新区开发，加快推进上汽通用五菱、柳工等企业园区建设。不断增强县区工业园区承载功能，努力构筑布局合理、相互促进、各具特色的园区发展新格局。今年，重点监测工业园区规模以上工业产值要达到1000亿元以上。

　　二、深入实施城乡统筹发展新格局战略，全面加快城乡一体化进程

　　实施城乡统筹升级规划，以工促农、以城带乡，实现工业化与城镇化良性互动，努力构建城乡统筹协调发展新格局。

　　加大统筹城乡发展力度。推进城乡规划管理一体化，进一步完善县城规划，启动重点乡镇产业规划，加快六县村庄规划并确保年内覆盖率达60%以上。完成雒容行政区划调整。加强对柳江、鹿寨两县规划工作指导，着力打造柳江、鹿寨两个城市副中心。深入实施城乡互动大通道工程，加快三江至柳州高速公路建设，争取开工沙塘至沙浦道路改造，推进拉堡经进德至新兴道路建设，强化“一小时经济圈”、“两小时经济带”布局框架。加快柳北城镇带建设，扎实推进柳北区石碑坪镇城乡建设用地增减挂钩试点，对主要节点城镇及近郊城镇实施城镇化改造，提升城镇综合承载能力。

　　深入实施“十大农业工程”。年内计划投资11.24亿元。继续实施良种扩繁工程和产业基地工程，加快甘蔗新品种、油茶良种、无病毒柑橘苗木、良种生猪、奶水牛的繁育和引进，扩大油茶、毛竹、超级稻、蔬菜、葡萄、食用菌、特色水产等产业基地规模。新造油茶4万亩,油茶低改11.6万亩；新造杉竹混交林3万亩,毛竹低改5万亩；实施人工造林28万亩。推进“金太阳”新农村建设暨生态农业示范区项目，开工建设柳州农业产业博览园，实现现代生态农业旅游工程和现代农业科技示范园工程提速。实施40个乡镇农业五站改造，完成柳州农机大市场暨农机科技产业园规划设计。推进100个新农村试点建设“三项会战”。实施整村推进贫困地区集中连片开发，扎实推进70个村屯的扶贫攻坚。

　　进一步改善农村生产生活条件。加大农村公路交通基础设施建设力度，确保农村公路养护、水毁和安保的资金投入，推进农村公路建设可持续发展。启动落久水利枢纽建设，加快推进洋溪水利枢纽前期工作。实施农田水利化工程，积极推进小（二）型水库除险加固和中小河流治理，着力抓好小型农田水利建设，推进一批中小型灌区节水工程改造。新建农村饮水安全工程200处，解决15万农村人口饮水问题。全面完成大石山区人畜饮水工程建设大会战任务。继续推进农村电网改造、城乡风貌改造和农村危房改造。启动实施农户万元增收计划，促进农民持续增收。

　　大力发展现代农业。在2010年基础上继续大幅度增加农业投入。实施粮食安全工程，确保粮食产量稳定在80万吨以上。加快糖料蔗、水果、桑蚕、蔬菜、食用菌、茶叶等优势产业发展，积极推进品种结构优化升级。加快农业产业化进程，力争新增市级以上龙头企业5家，新增农民专业合作社50家。实施农机化创新示范工程，推进水稻生产全程机械化，突破甘蔗机械化收获瓶颈。统筹推进新一轮“菜篮子”工程，推广设施农业和标准化生产技术，并在灌溉设施、品种开发、规模养殖等方面给予补贴，加快建设蔬菜、肉、蛋、奶、水产品标准化生产基地和产业区。积极创建水产健康养殖示范场，推进生猪、优质家禽家畜养殖基地建设，加快畜禽养殖加工一体化。

　　加快县域经济发展。优化市县工业布局，加快汽配、机械、电力、建材、化工等产业向县域聚集，着力打造一批百亿元产业集中区。加快县域特色产业发展，做大做优制糖、茧丝绸、制药、竹木加工、茶叶等产业，形成各具特色的县域产业体系。

　 三、全力加快服务业发展，进一步提升城市综合服务功能

　　全面落实自治区关于加快服务业发展的政策措施，着力优化服务业结构，努力提升服务业水平，为“聚集人气，做大城市”提供强力支撑。

　　促进与扩大城乡居民消费。努力提高城乡居民收入，增强居民消费能力。积极培育旅游、健身、文化、娱乐等新的消费热点。认真组织好重点消费品的集中促销活动。积极挖掘和整理柳州菜系，扩大餐饮服务业消费。继续实施“万村千乡市场工程”、“新农村现代物流工程” ，年内完成27个乡镇集贸市场升级改造，新建与改造50家社区微型超市，不断改善城乡消费环境。今年，争取全市社会消费品零售总额增长18%以上。继续加强市场保障和价格稳定工作，整顿和规范市场价格秩序。加大房地产价格监测和市场调控力度，保持房地产业平稳健康发展。

　　加快商贸物流业发展。继续实施“十大商贸工程”，加快钢铁、化工、农产品、粮食四大千亩物流园建设。开工建设东盟国际物流园、东盟小商品生产加工配送中心等物流项目，启动柳东新区物流城建设。实施汽车南站—金鹅市场综合改造，启动南方花鸟市场等专业市场建设。完善片区商业布局，提升城市商业档次，加快新风时代购物广场和风情港、地王财富购物中心建设，启动河东购物中心项目。培育一批大型现代商贸企业，加快推进工贸与百货合并重组，着力打造商业零售旗舰，创建服务企业品牌。加快发展第三方物流，重点发展汽车、冶金等生产性专业物流，促进制造业与物流业联动发展。

　　加强“旅游名城”建设。深入实施“十大旅游工程”。加快窑埠古镇、三门江森林公园及环江带旅游等项目建设，按精品公园对龙潭公园进行大规模改造，启动红花谷湿地公园规划及项目申报工作，完善和提升“百里柳江”都市休闲旅游、“百里风情”民俗风情旅游和环都市乡村旅游三大产品集群。全面落实“旅游名城”奖励政策，推动A级景区和工农业旅游示范点创建，促进星级酒店建设与升级，扩大旅游区域合作和市场宣传，加大引客入柳力度。完善柳州旅游标识系统。结合高速公路、高速铁路建设，加快重点旅游县景区规划建设，继续推进三江民族风情园、柳城刘三姐影视文化城等项目，启动融水元宝山景区项目，加快融安红茶沟景区项目前期工作，不断提升“风情柳州”旅游品牌。

　　大力发展新兴服务业。加快发展会展、商务、金融、信息、审计、评估等生产性服务业，积极引进和发展各类专业与中介服务机构，培育发展会展经济、总部经济和楼宇经济，不断提升城市服务品质和影响力。大力发展医疗卫生、物业服务、家政服务、社区服务、养老服务等面向民生的生活性服务业，努力满足城镇化和人口老龄化的新需求。

　　四、突出承载能力和生态环境建设，着力打造超大城市

　　按照建设超大城市的战略构想和“一心两城、沿江发展、重点向东”的城市发展方向来打基础拉框架，进一步提升城市综合承载能力，加快城市升级转型。

　　积极拓展对外交通。加快湘桂铁路扩能改造，确保柳南城际铁路年内通车，力争柳肇铁路开工建设。积极做好柳韶铁路前期工作。抓紧实施柳州火车站房及广场扩建工程和东站货场搬迁工程，积极做好柳东新区铁路货场专用线推进工作。继续推进阳朔至鹿寨、柳州至武宣等高速公路建设，力争开工柳州至梧州高速公路，积极推进柳州至南宁第二高速公路前期工作。加快黄金水道项目建设，竣工鹧鸪江码头和红花水电站至象州石龙三江口航道整治工程，加快柳州港各作业区项目前期或建设工作，积极推动大藤峡水利枢纽开工建设。加快白莲机场扩建，力争开辟更多新航线。

　　以科学规划引领城市建设。完善片区建设规划，以广西柳州汽车城规划为基础，加快新区总体规划调整，完善新区的控制性详规和专项规划。强化专项规划，统筹安排教育、医疗、市政等公共服务设施的规划布局和建设。完成县、乡镇土地利用总体规划修编。加强土地节约集约利用管理，切实做好征地拆迁和用地供应工作，加大违法建筑、违法占地治理力度。全面完成我市第二次土地调查工作。

　　加大城市基础设施建设。以“十大建设工程”为载体，加快宜居城市建设，市政重点工程投资155亿元以上。推进无障碍城市建设。完善城市道路交通网络，启动河东片区交通枢纽建设，加大道路主要节点和瓶颈道路改造力度，继续疏通支路巷道，缓解城市交通压力。加快城市快速环道建设，争取全线通车。扎实推进“城中村”改造和旧城改造，重点抓好桂柳路和静兰、古亭山片区改造，尽快把旧城新区连片，打造好柳州东大门。全面推进“柳东新区十大项目”，加快市区至新区快速公交系统（BRT）等项目进度，加快新区市政配套设施、供电基础和公用服务设施建设。

　　全力争创国家森林城市和国家环境保护模范城市。推进生态市及生态县建设。实施“绿满龙城”工程，继续开展“百万人种百万棵树”活动，让森林走进城市，让柳州拥抱森林。加强园林绿化建设，城市绿化投入经费在上年基础上翻一番，达2亿元。完成马鹿山公园二期，加快雀山等公园提升改造和10个小游园建设。继续推进道路美化和市区石山绿化，全面推进百里柳江两岸园林绿化景观建设。突出生态环保建设，强化环境监测预警和执法监督，确保污染企业达标排放，确保市、县污水及生活垃圾处理设施正常运转；强化环境综合治理，按国家要求把氮氧化物和氨氮纳入总量控制；逐步加强农村环境治理和保护，加大水源林保护和柳江上游生态保护力度，确保柳江水质和市区空气质量持续改善。今年，要力争成为国家森林城市和国家环境保护模范城市。

　　提升城市管理水平。结合“六城同创”，加强柳江河道治理，深入推进城乡清洁工程和市容市貌综合整治，规范建筑施工工地管理，严格建筑垃圾密闭化运输，继续完善公厕、垃圾中转站等环卫配套设施建设。 开展城市道路广告整治，规范道路（街道）路牌设置，坚决取缔路牌广告，严格控制道路广告。实施交通疏导工程和“文明交通行动计划”，开展规范管理电动车专项整治行动，着力解决交通混乱、拥堵和停车难问题。加强城区数字城管二级中心建设，实现与“天网工程”信息共享；加强城管队伍建设和管理，做到公正文明执法，努力构建和谐城管。

　 五、着力改善和保障民生，让人民群众分享更多的改革发展成果

　　坚持把改善民生作为一切工作的出发点和落脚点，深入实施“十大民生改善工程”，逐步建立居民收入与经济增长、城乡低保与物价上涨等联动机制，确保城乡人民生活水平与经济社会发展水平同步提高。今年，要继续为民办好20件实事。

　　促进创业带动就业。不断完善组织机构、政策、服务、培训和考核五大创业体系，全面落实创业扶持政策，优化全民创业环境，确保我市通过国家级创业型城市验收。实施就业援助工程，加大对就业困难群体的帮扶力度，重点帮助高校毕业生、下岗失业人员、残疾人和返乡农民工解决就业问题，做好“零就业家庭”和“经济困难毕业生家庭”的岗位援助工作。积极统筹城乡就业，加大农村劳动力培训和转移力度。确保城镇新增就业人数5万人，实现转移就业5万人。

　　进一步完善社会保障体系。继续强化社会保险扩面征缴工作，确保各项社会保险待遇按时足额发放。重点抓好融水、鹿寨开展第二批新型农村社会养老保险试点工作。按照国家部署继续提高企业退休人员基本养老金。落实调整后的职工医保新政策，扎实做好居民医保参保工作。完善与经济发展水平挂钩的最低工资制度。建立城乡居民最低生活保障标准自然增长机制，适时调整城乡低保标准。继续完善城乡社会救助体系，加快市社会福利救助服务中心建设。继续实施关爱残疾人“阳光计划”。加强备灾救灾及困难救济工作，进一步完善各级自然灾害应急预案。切实做好库区移民工作。

　　推进重点民生工程建设。进一步扩大改制企业职工危旧房集中区改造覆盖面，年内投资5.6亿元，实施改造79万平方米（含续建），竣工21.4万平方米。保障性安居工程新开工建设经济适用房1700套13.6万平方米，新建廉租房900套4.5万平方米。引导和支持社会力量参与公共租赁住房建设，着力解决中等偏下收入家庭及新生劳动者的住房困难，力争年内新建（筹集）公共租赁房1800套。启动新一轮村寨防火改造工程，开展融水县30-50户村屯的防火改造试点。继续实施少数民族村寨火灾统保工程，积极推进全市农村住房统一保险工作。切实提升九年义务教育农村学生免费午餐质量，规范经费管理。继续实施老有所养工程，加快推进老年人休养集中区建设，进一步扩大社区居家养老试点。

　　切实维护社会稳定。深入创建“平安柳州”，加强社会治安综合治理，完成“天网工程”三期建设，使治安监控点增至2300个，以加强对学校、幼儿园等重要场所、重点区域和关键节点的监控，让城市更安全、市民更安心。切实做好农村“三大纠纷”调处，健全信访工作长效机制，持续开展领导干部公开大接访活动，妥善化解社会矛盾。继续开展食品药品安全专项整治，逐步完善检验监测体系。强化安全生产监督，促进安全生产形势持续稳定好转。

　　六、深化改革，扩大开放，增强科学发展的动力与活力

　　坚持以开放促发展，以改革促活力，全面加快发展方式转变和经济升级转型。

　　深入推进各项改革。继续完善国有投融资平台建设，不断增强平台公司投融资能力。继续推进柳化增资扩股与战略扩张，支持柳州银行加快跨区域扩张，推进农工商、鹧鸪江园艺场等企业改制。全面加快柳州医药、桂中海迅等企业上市步伐，支持上市公司再融资和企业融资租赁、发行债券。放手发展民营经济，全面贯彻落实自治区关于促进民营经济发展和发展微型企业的政策措施，努力拓宽民营经济发展空间，进一步激活民间资本。稳妥实施省直管县财政改革。积极推进村镇银行等农村新型金融机构试点。协调推进农村综合改革；全面完成集体林权制度主体改革；深入推进柳江县扩权强县改革试点和扩权强镇试点。积极探索户籍制度改革，适当放宽城市落户条件，积极引导农民向城市、城镇转移。

　　加大招商引资力度。紧紧围绕“三年四千亿，工业再翻番”战略和系列十大工程，以建设广西柳州汽车城为契机，突出产业链招商和招大引强两大重点，加强项目策划和运作，不断完善招商机制，创新招商模式，力争在引进大型产业项目和推进“央企入柳”、“引金入柳”等方面实现新突破，确保引进市外境内资金400亿元以上，利用外资2.8亿美元以上。要强化招商引资大兑现工作，确保签约项目按期落地。

　　促进外经外贸平稳发展。完善促进外贸出口政策，优化出口结构，转变外贸发展方式，确保全市进出口总值增长15%以上，努力将外贸出口恢复到金融危机前水平。切实加大对南亚、南美、俄罗斯等新兴市场的开拓力度，继续举办海外工业品展销会，举办首届中国—东盟汽车、工程机械及零部件博览会，拓展外贸出口新的增长点。加大“走出去”力度，支持企业在境外建立办事处、营销中心和售后服务网络；鼓励有条件的企业到境外投资，开展资源、能源合作，稳定进出口渠道，加快企业和产业国际化步伐。大力发展加工贸易，启动综合保税区（出口加工区）申报工作。加强口岸基础设施建设，进一步优化通关环境。

　 七、统筹社会事业发展，顺应人民群众过上美好生活的新期待

　　切实加大社会事业投入力度，努力满足人民群众日益增长的公共服务和精神文化需求。今年，市财政安排社会事业资金17.7亿元，增长30.6%。

　　深入实施科教兴市和人才强市战略。密切产学研衔接，增进与国内知名高校院所的交流与合作，进一步促进科技成果转化。实施知识产权战略，加强知识产权创造、运用和保护。广泛开展科普活动，积极普及节能、节水和环保知识与技术，推进市科技馆、青少年宫建设。今年，力争第六次成为“全国科技进步先进市”，确保各县区通过全国科技进步考核。实施人才聚集工程。全面落实人才奖励政策，积极鼓励和引进海内外高层次人才、紧缺专业人才到柳工作或创业；继续选送优秀青年赴海外深造；认真实施自治区“八桂学者制度”和“特聘专家制度”、市政府顾问和特殊津贴专家制度；继续抓好人才小高地、博士后工作站建设，不断强化我市人才支撑体系。

　　优先发展教育。以“十大教育工程”为载体，扎实推进教育布局结构调整，统筹各级各类教育协调发展。优化教育资源配置，稳步推进农村义务教育集中办学，不断缩小城乡义务教育差距。全面实施“学前教育三年行动计划”，通过政府主导、社会参与、公办民办并举，大力发展公办幼儿园，扶持发展民办尤其是村级民办幼儿园，年内市区新建4所公办幼儿园，每县至少新增2所公办乡镇中心幼儿园；鼓励有条件的学校、幼儿园实施校车制度，努力探索一条学前教育就近入园、农村义务教育相对集中寄宿的办学模式，从根本上解决农村留守儿童的教育和管护问题。继续加大校舍安全工程建设力度，确保完成自治区下达的年度建设任务。全面加快市、县特殊教育学校建设。以建设职教园和国家民族地区职业教育综合改革试验区为依托，提升职业教育品牌，努力打造西南地区最大的现代职业教育基地。重视发展高等教育，加快广西工学院与柳州医专合并组建综合性大学步伐，积极筹建汽车类高等院校。

　　繁荣发展文化、体育事业。继续推进“十大文化建设工程”，全面加快工业博物馆、白莲洞古人类遗址博物馆、军事博物园等项目建设。积极推进列为自治区百家博物馆项目建设。不断完善公共文化服务体系，新建9个乡镇文化站、53个村级公共服务中心、12个社区文化活动中心、80个农家书屋和200个街道、社区阅报栏，在柳东新区筹建集图书、音像制品发行和物流配送为一体的大型图书城。大力发展文化产业，继续推进文化体制改革，整合文化资源，成立市文化产业发展集团，积极探索有柳州特色的文化发展道路。繁荣发展广播电视、新闻出版和文学艺术创作，抓好精品剧目运作和引进，培育文化消费市场，确保新创排大剧首演和电视剧《刘三姐》首播。合理组织水上大舞台公益性演出，积极开展丰富多彩的群众文化活动。加强非物质文化遗产与文博资源的保护和利用。认真开展第二轮修志工作。积极发展体育事业，依托百里柳江水上资源，扩大龙舟大赛等特色传统赛事的影响力，继续承办世界水上极速运动大赛等国际性体育赛事，策划举办“国际水上狂欢节”。继续完善城乡体育基础设施，深入贯彻落实《全民健身条例》，推动全民健身活动全面开展。

　　加快发展医疗卫生、人口与计生事业。深化医疗卫生体制改革。完善新农合制度，提高新农合保障水平，不断扩大新农合覆盖面和受益面。推进国家基本药物制度和基层医疗机构综合改革，稳步推进公立医院改革试点。进一步完善公共卫生和基本医疗卫生服务体系，继续抓好基本和重大公共卫生服务项目，逐步提高城乡公共卫生服务水平和质量，促进公共卫生服务均等化。着力解决社区卫生服务中心的用房用地问题。着力抓好艾滋病等重大传染病的防控工作。强化疾病预防控制，深化人口计生综合改革，大力推进婚育新风进万家活动，实施优生促进工程，改善出生人口素质，不断提高人口与计生工作水平。

　　着力加强精神文明建设。全力争创全国文明城市和全国卫生城市。深入开展群众性精神文明创建活动，加强社会主义核心价值体系建设。实施文明素质提升计划，提升公民道德水准，培养全社会良好的道德风尚。继续加强民族团结宣传教育，深入开展民族团结进步创建活动。认真做好国防动员和民兵预备役工作，扎实推进双拥共建，不断巩固军政军民团结，确保夺取“全国双拥模范城”七连冠。加强未成年人保护，发展妇女儿童事业。大力倡导志愿服务，鼓励企业和市民积极参与慈善与公益事业。

　　八、加强政府自身建设，努力打造人民满意政府

　　深入推进“十大党建工程”，以党的建设引领和推动政府建设，不断提升政府依法行政能力和公共服务水平。

　　进一步优化政务环境。切实做好政府规范性文件的审查、清理，继续开展行政审批事项的清理、规范工作。加强市、县(区)、乡镇(街道)、村（社区）四级政务服务网络建设，乡镇（街道）设立政务服务中心达50%以上。深入推进机关行政效能建设，强化政务督查和效能监察。继续加强绩效考评，强化绩效管理理念，提升绩效管理水平。持续开展行动学习，推进学习型、创新型政府建设，努力打造具有柳州特色的行政文化。

　　加强民主法治建设。各级政府和政府部门要自觉接受人大及其常委会的监督、政协的民主监督，严格执行人大及其常委会的决议、决定，认真办理人大代表的议案、建议和政协委员提案，广泛听取各民主党派、工商联、无党派人士和人民团体的意见。全面推进《法治柳州建设规划》，努力创建法治城市。启动“六五”普法，强化依法行政。积极推进政务公开、厂务公开和村务公开，不断健全基层群众依法自治机制。

　　加强党风廉政建设。继续建立健全惩治和预防腐败体系，全面落实党风廉政建设责任制，强化行政监察和审计监督，严格责任追究，确保行政权力依法、公正、有效运行。坚持标本兼治、纠建并举，深入开展损害群众利益不正之风专项治理，不断完善防治不正之风长效机制。

　　各位代表，回顾过去，我们将“十一五”蓝图变成了美好现实；展望未来，我们站在了“十二五”的新起点上。让我们紧密团结在以胡锦涛同志为总书记的党中央周围，高举邓小平理论和“三个代表”重要思想伟大旗帜，深入贯彻落实科学发展观，在市委的正确领导下，锐意进取，开拓创新，扎实向上，奋发有为，为实现“十二五”的良好开局和建设更加美好的柳州而努力奋斗！
